# Disposable gold nanoparticle functionalized and bare screen-printed electrodes for potentiometric determination of trazodone hydrochloride in pure form and pharmaceutical preparations†

**DOI:** 10.1039/c8ra00745d

**Published:** 2018-03-23

**Authors:** Fathy M. Salama, Khalid A. Attia, Ragab A. Said, Ahmed El-Olemy, Ahmed M. Abdel-raoof

**Affiliations:** Pharmaceutical Analytical Chemistry Department, Faculty of Pharmacy, Al-Azhar University 11751 Nasr City Cairo Egypt Ahmedmeetyazeed79@yahoo.com

## Abstract

In the present study, screen-printed electrodes unmodified and chemically modified with gold nanoparticles were used as sensitive electrochemical sensors for the determination of trazodone hydrochloride. The sensors were based on the use of a tetraphenylborate ion association complex as an electroactive material in screen-printed electrodes with dioctyl phthalate (DOP) as a solvent mediator modified with gold nanoparticles which improve the electrode conductivity and enhance the surface area. The sensors displayed a stable response for 5, 6 and 7 months with a reproducible potential and linear response over the concentration range 1 × 10^−5^–1 × 10^−2^ mol L^−1^ at 25 ± 1 °C with Nernstian slopes of 57.50 ± 0.66, 58.30 ± 0.45 and 59.05 ± 0.58 mV per decade and detection limits of 7.9 × 10^−6^, 7.6 × 10^−6^ and 6.8 × 10^−6^ mol L^−1^ for sensor 1, 2 and 3 respectively. The analytical performance of the screen printed electrodes in terms of selectivity coefficients for trazodone hydrochloride relative to the number of potentially interfering substances was investigated. The proposed method has been applied successfully for the analysis of the drug in its pure and dosage forms and there is no interference from any common pharmaceutical additives.

## Abstract 2

In the present study, screen-printed electrodes unmodified and chemically modified with gold nanoparticles were used as sensitive electrochemical sensors for the determination of trazodone hydrochloride.

## Graphical abstract

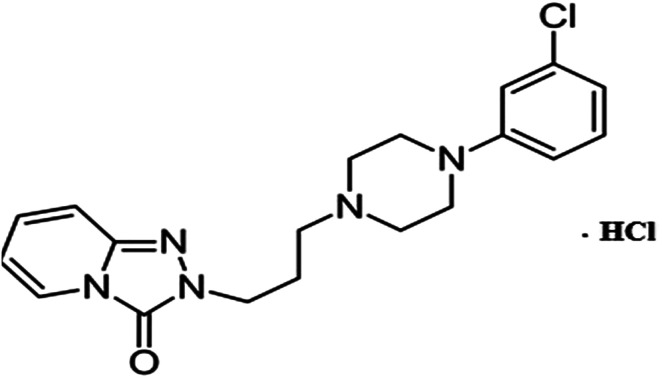

## Introduction

Trazodone hydrochloride (TZ) (2-{3-[4-(3-chlorophenyl)piperazin-1-yl]propyl}-2*H*,3*H*-[1,2,4]triazolo[4,3-*a*]pyridin-3-one) is a well-known chemical compound that is used as an antidepressant and belongs to a class of selective serotonin reuptake inhibitor (SARI).^1^ TZ dissolves in methanol at 25 mg mL^−1^ to yield a clear, colorless solution. It also dissolves in water at 50 mg mL^−1^, with heating, to yield a hazy colorless solution.^1^ The official method for the analysis of trazodone hydrochloride is based on potentiometric non-aqueous titration with perchloric acid^2^ and HPLC using an octadecylsilane column and methanol–0.01 M ammonium phosphate buffer at pH 6.0 (60 : 40) as the mobile phase.^3^ A literature survey revealed that several techniques have been developed for the quantitative determination of trazodone hydrochloride in pharmaceutical formulations, including spectrophotometric methods,^4–9^ potentiometric methods,^10,11^ voltammetry,^12–15^ and different chromatographic methods including HPLC,^3,16–21^ capillary gas chromatography,^22^ high-performance liquid chromatography-tandem mass spectrometry,^23,24^ and high performance thin layer chromatography.^25^ In addition, spectrofluorimetric methods have been reported.^26,27^

**Fig. 1:**
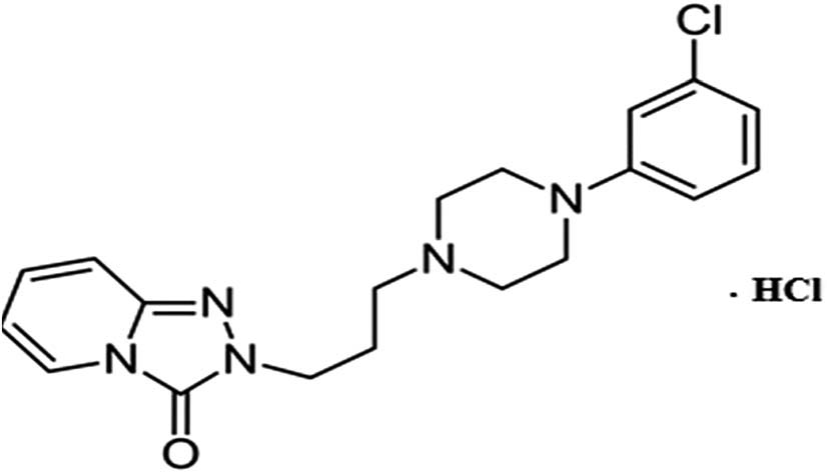
Structural formula of trazodone hydrochloride.

In the present work, we prepare simple, sensitive and selective bare, functionalized and gold nanoparticle screen printed electrodes to quantify trazodone hydrochloride in a variety of samples. The advantages of screen-printed electrodes include the diverse range of paste modifications available and their convenience of handling. The constructed electrodes were optimized according to the IUPAC recommendations and were successfully applied as sensors to determine trazodone hydrochloride in pure and pharmaceutical forms (Fig. 1).

## Experimental

### Apparatus

The potentiometric measurements were carried out using the pH-meter Jenway 3510 (England) with an Ag/AgCl reference electrode in combination with the drug sensor, a Benchtop pH meter (BP 3001), Singapore, equipped with a pH electrode with a built-in temperature sensor, and a Bandelin sonorex, Rx 510 S, with a magnetic stirrer (Hungary). A Shimadzu UV-vis 1650 spectrophotometer (Japan), a hot plate (Torrey Pines Scientific, USA) and a bench top centrifuge (TDL-60B) with max. speed 6000 rpm (Hunan, China, Mainland) were also used. The gold nanoparticle samples were examined by scanning electron microscopy (SEM) (JEOL JSM-5500LV, JEOL Ltd, Japan) using the low vacuum mode, the gold coating was examined using an SPI-model sputters coater and a JEOL-1010 transmission electron microscope, Japan, at 80 kV was employed for the transmission electron microscopy (TEM) examinations at the Regional Center of Mycology and Biotechnology, Cairo, Egypt.

### Reagents

All reagents were of analytical grade and double distilled water was used throughout the experiments. Trazodone hydrochloride powder was kindly supplied by Egyptian International Pharmaceutical Industries Company (Eipico), 10th of Ramadan City, Egypt. Its purity was 99.45 ± 0.25% (batch no. A0967913). Trittico® tablets, labeled to contain 100 mg of trazodone hydrochloride per tablet and manufactured by Egyptian International Pharmaceutical Industries Company (Eipico), 10th of Ramadan City, batch no. 1602039 were purchased from the local market. Dioctylphthalate (DOP) was obtained from Fluka, Germany. Sodium tetraphenyl borate (TPB), polyvinylchloride (PVC, relatively high molecular weight), multi-walled carbon nanotube powder (carbon >95.0%, O.D. × *L* 6–9 nm × 5 μm), hydrogen tetrachloroaurate (HAuCl_4_·3H_2_O), trisodium citrate and cyclohexanone were obtained from (Sigma-Aldrich, Germany). Sodium hydroxide, hydrochloric acid (37% w/w), nitric acid (55.5% w/w), sulfuric (98% w/w) and acetone were obtained from El-Nasr Company, Egypt.

### Standard drug solutions

Stock drug solution (10^−2^ mol L^−1^) was prepared by dissolving 408.32 mg of trazodone hydrochloride in double distilled water and the volume was brought up to 100 mL with the same solvent. Other solutions (1.0 × 10^−3^–1 × 10^−7^ mol L^−1^) were prepared by serial dilution from the stock solution.

### Preparation of the ion association complex

The electroactive material, a trazodone–tetraphenyl borate ion pair (TZ–TPB), was prepared by mixing 50 mL of both 1 × 10^−2^ mol L^−1^ TZ and TPB solutions. The resulting precipitate was filtered off through Whatman filter paper no. 42, washed with cold water several times, dried at room temperature and ground to fine powder.

### Preparation of functionalized multi-walled carbon nanotubes

1.5 g of MWCNTs was refluxed with H_2_SO_4_ + HNO_3_ (3 : 1) at 55 °C for 12 hours. Then the reaction mixture was stirred at 40 °C for 12 hours, diluted three times with distilled water and filtered using a centrifuge machine at 4000 rpm. The process of centrifugation and washing off with distilled water was repeated until neutral pH was achieved. Then the sample was dried in a vacuum oven at 60 °C for 24 h to give carboxylated MWCNTs (MWCNT-COOH). This leads to the opening of the caps of the MWCNTs and formation of functionalized carbon nanotubes.^28,29^

### Procedure for the preparation of citrate-capped gold nanoparticles

Au NPs (gold nanoparticles) were prepared by a sodium citrate reduction method.^30–33^ To a 150 mL beaker, 4 mL of 1% HAuCl_4_ and about 100 mL of water were added and the solution was heated to 95 °C. 10 mL of 1% sodium citrate solution was added drop by drop while the solution was vigorously stirred. The solution was kept at 95 °C for 10 min. When the color of the solution changed to bright purple, the solution was allowed to cool to room temperature and transferred into a 100 mL volumetric flask, diluted to the mark with water and mixed well. The average size of the prepared Au NPs was about 12.20 ± 2.53 nm, which was estimated from the TEM image.

### Sensor construction

The printing process involved several stages including silk screen manufacturing, homemade ink synthesis, paste preparation and finally, printing the electrode through the silk screen. A screen is made of a silk fabric textile (mesh count 36) which was prestressed to an aluminum frame (30 cm × 40 cm) and the screen was coated with a photographic chemical solution that is sensitive to light known as the photographic emulsion, which was spread onto the fabric silk screen. This emulsion-coated screen was dried at 50 °C for 20 min and through several steps an array of 12 electrodes was printed on a PVC film. The required final electrode shape consisted of 3 × 4 electrodes, each 5 mm × 35 mm, and was printed twice on a polyester sheet and then this photographic positive sheet was used to produce the electrode template. The photographic positive containing the electrode shape was taped to the emulsion-coated screen and exposed to UV light for 13–15 min. When the light is passing through the screen, the open area of the screen called the stencil will be developed for only the area darkened by the film positive after washing with water, then the screen will have dried and is ready for printing and the ink is placed in the screen and driven through the stencil with a “squeegee”, thus creating a printed image. The homemade printing ink was prepared as described in detail elsewhere^34–36^ by thoroughly mixing 50 mg of the TZ–TPB ionophore with 0.6 g of DOP, 2 g of PVC solution (8% in cyclohexanone–acetone mixture 1 : 1) and 1 g of carbon nanotube powder (sensor 1). A functionalized paste was prepared in a similar manner, except that the bare carbon nanotube powder was replaced with the desired weight of carboxylated carbon nanotube powder (sensor 2) and 1.5 mL gold nanoparticles was added to sensor 2 to prepare a functionalized modified screen printed electrode with gold nanoparticles (sensor 3) to obtain different compositions, as shown in Table 1. The working electrodes were printed on the plain X-ray sheet using a plastic squeegee and cured at 60 °C for 60 minutes to evaporate the residual solvent. The working electrode was insulated leaving the working area (5 mm × 5 mm) for electrical contact and an equal area on the other side for dipping in the sample solution. Fabricated SPEs were used directly in measurements after three calibrations, which served as a preconditioning process.

**Table 1:** The optimal matrix paste compositions of the different SPE sensors

Sensor type	Composition				
	Ion pair (mg)	Carbon nanotube (mg)	Functionalized carbon nanotube (mg)	Gold nanoparticles (mL)	Plasticiser (mg)
Sensor 1	50	1000	—	—	600
Sensor 2	50	—	1000	—	600
Sensor 3	50	—	1000	1.5	600

### Potentiometric measurements

The fabricated sensors were calibrated by transferring 25 mL aliquots of concentration 10^−7^ to 10^−2^ mol L^−1^ into the measuring cell followed by dipping the working electrode in combination with the reference electrode into these solutions. Calibration graphs were obtained by plotting the observed potential *versus* the logarithm of the trazodonium ion activity and the electrode performances were evaluated according to IUPAC recommendations.^37^

### Pharmaceutical sample analysis

Ten Trittico® tablets were accurately weighed and finely powdered, then a quantity equivalent to 408.32 mg of trazodone hydrochloride was shaken three times with 25 mL of water for 15 minutes, which was then filtered into a 100 mL volumetric flask and then the volume was adjusted to the mark with water to obtain a concentration of 10^−2^ mol L^−1^. The solution was analyzed using the procedure described under the proposed method. The standard addition technique was applied by adding certain known volumes (at 10^−2^ mol L^−1^) of pure drug solution to 25 mL aliquot samples (10^−5^–10^−3^ mol L^−1^) of the pure sample.^38–41^ The change in mV reading was recorded for each increment and used to calculate the concentration of the drug in the sample solution using the following equation:
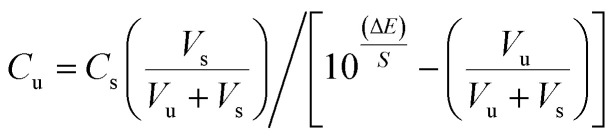
where *C*_u_ and *V*_u_ are the concentration and volume of solution to be determined, respectively, *V*_s_ and *C*_s_ are the volume and concentration of the standard solution added to the sample under testing, respectively, Δ*E* (*E*_2_ − *E*_1_) is the change in potential caused by the addition, where *E*_1_ = electrode potential (mV) in the pure sample solution and *E*_2_ = electrode potential after the addition of the standard, and *S* is the slope of the calibration graph.

## Results and discussion

### Composition of the electrodes

**Fig. 2:**
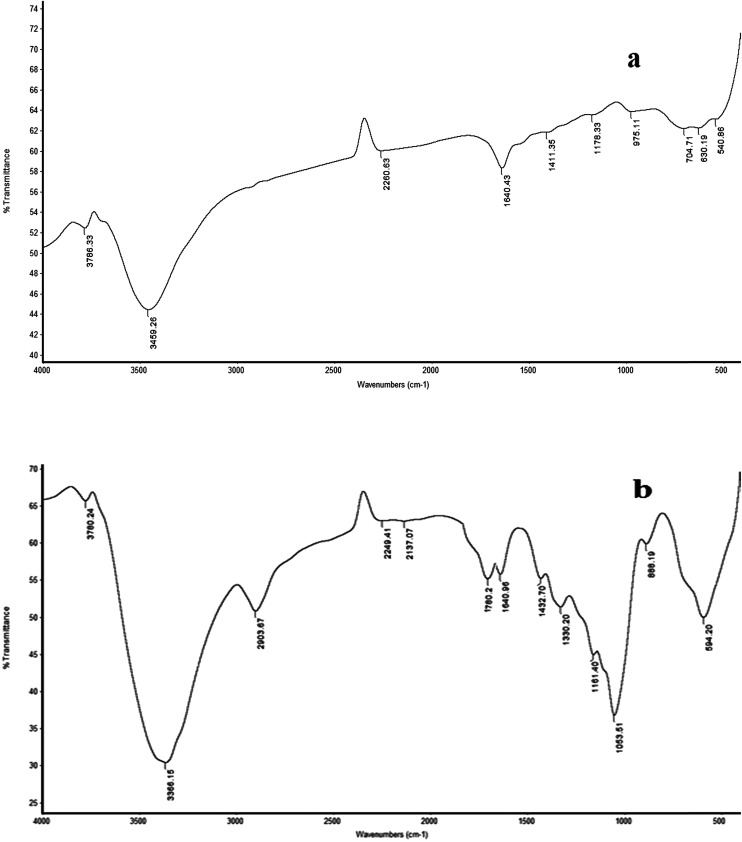
FT-IR spectra of the (a) MWCNTs and (b) MWCNT-COOH powder samples.

**Scheme 1:**
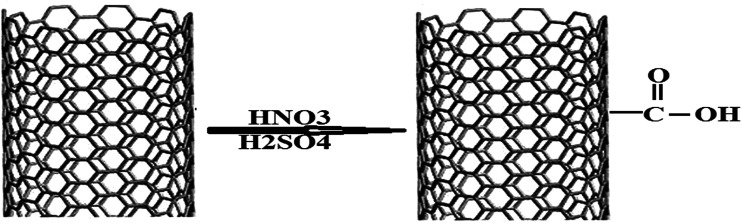
The reaction mechanism which takes place during the functionalization and sensing response.

The performance characteristics of a given ion selective electrode (ISE) based on an ion-pair depend to a large extent on its nature and composition, the properties of the plasticizer and especially any additives used, for example in sensor 2 we added carboxylated carbon nanotubes in which the carbon surfaces were oxidized by HNO_3_ and H_2_SO_4_ to increase the specific surface area and pore specific volume of the CNTs and their particle size decreased due to the fracture effect and the adsorption capacity onto the surface of the CNTs increased. Also, functionalization of CNTs overcomes the slow and incomplete recovery of a bare CNT-based sensor and improves interfacial interaction. The polar groups on the nanotube surface increase the adsorption affinity of the electron-donor or acceptor and enhance the sensing performance. Functionalized CNTs can be characterized by FT-IR as shown in Fig. 2(a and b) and represented by Scheme 1.

### Fourier transform infrared (FT-IR) studies

FTIR spectra of the MWCNTs show a broad peak at 3459 cm^−1^ which is characteristic of the OH stretch of the hydroxyl group while the carboxyl group on the surface of the bare CNTs could be due to partial oxidation of the surface of the CNTs during purification by the manufacturer. Fig. 2 shows the FT-IR spectra of the bare CNTs and functionalized CNTs. Fig. 2(b) shows other characteristic peaks of CNT–COOH at 1053 cm^−1^ (C–O), 1640 cm^−1^ (C=C) and 1760 cm^−1^ (C=O). As compared with the FT-IR spectrum of CNTs (Fig. 2(a)), the appearance of new peaks at 1760 and 1053 cm^−1^ in Fig. 2(b) is due to the stretching vibrations of the C=O and –C–O bonds in the carboxyl group (–COOH), respectively.

**Fig. 3:**
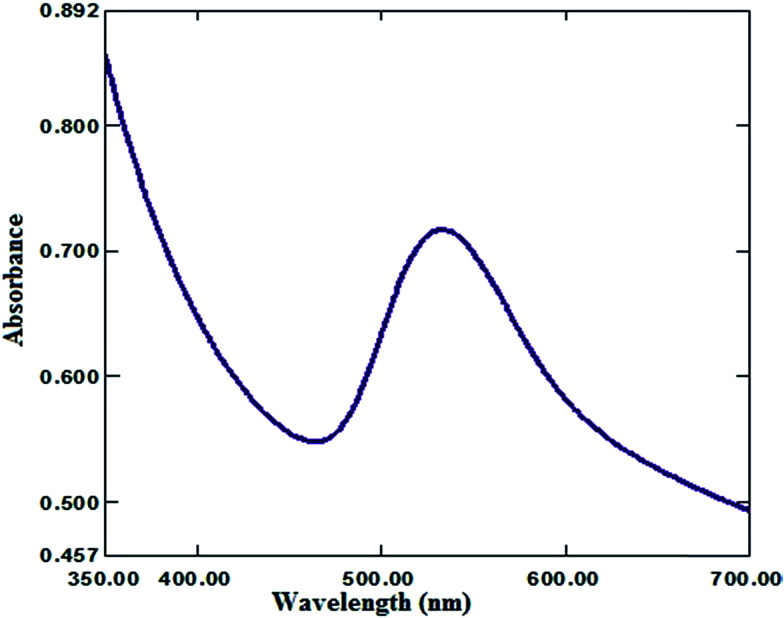
UV-visible spectrum of a freshly-prepared Au NP (12.20 ± 2.53 nm) suspension.

**Fig. 4:**
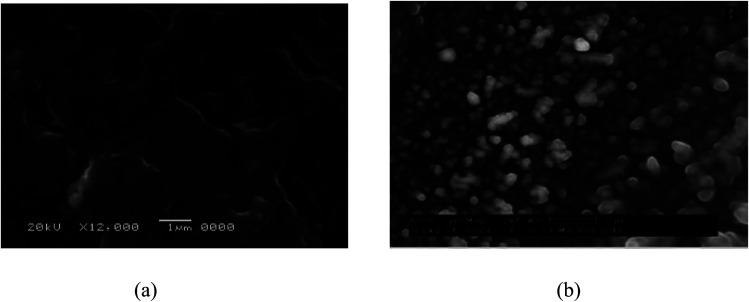
SEM images of the different electrodes: (a) the functionalized SPE and (b) the gold nanoparticle SPE.

**Fig. 5:**
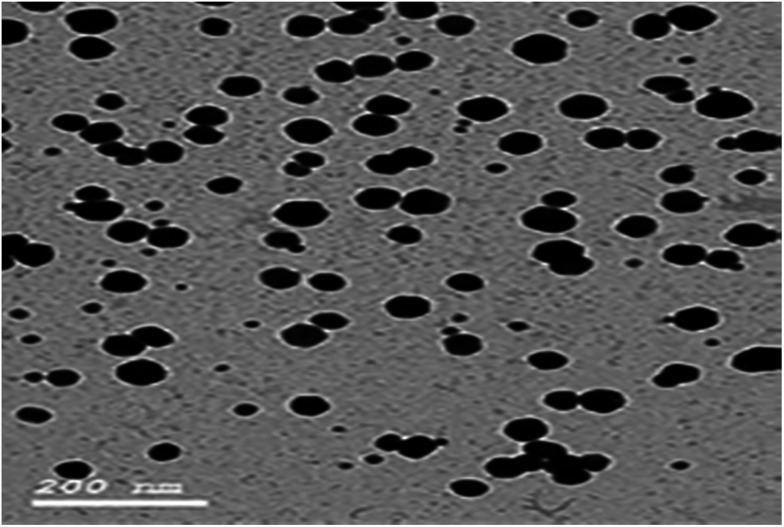
TEM image of the citrate-capped gold nanoparticles (Au NPs).

**Fig. 6:**
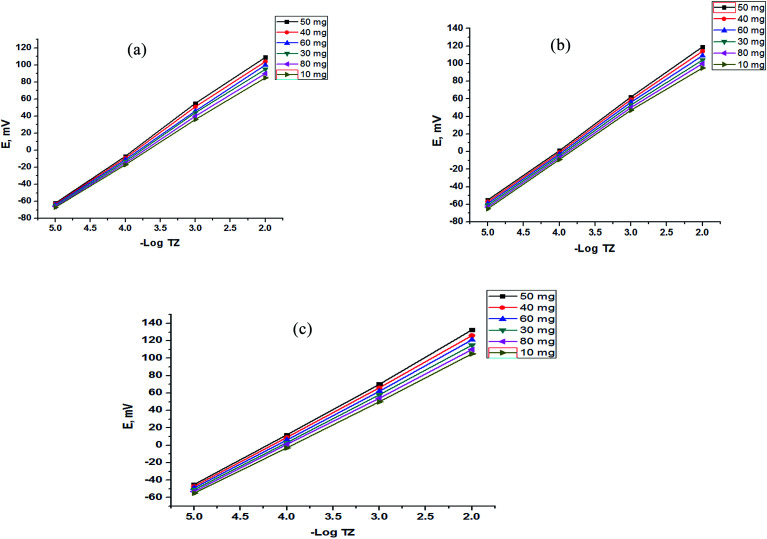
Effect of the content of the TZ–TPB ion pair for (a) a bare SPE, (b) a functionalized SPE and (c) a gold nanoparticle SPE.

Moreover, the addition of gold nanoparticles to the composition of sensor 2 will increase the performance of the SPE due to the ability of the carboxylate ion to interact with the gold nanoparticle surface; using this strategy gold nanoparticles can be densely adsorbed onto the sidewalls of multiwalled carbon nanotubes to improve their electrode conductivity and surface area.^42–44^ The characterization of gold nanoparticle formation was confirmed by UV-visible spectrophotometry, showing a maximum absorption at 531 nm as shown in Fig. 3. Gold nanoparticle formation was also confirmed by SEM (scanning electron microscopy) and TEM (transmission electron microscopy), as shown in Fig. 4 and 5 respectively, the ion pair content was studied and the results are shown in Fig. 6 and the optimum different electrode compositions are shown in Table 1.

### Sensor performance

**Fig. 7:**
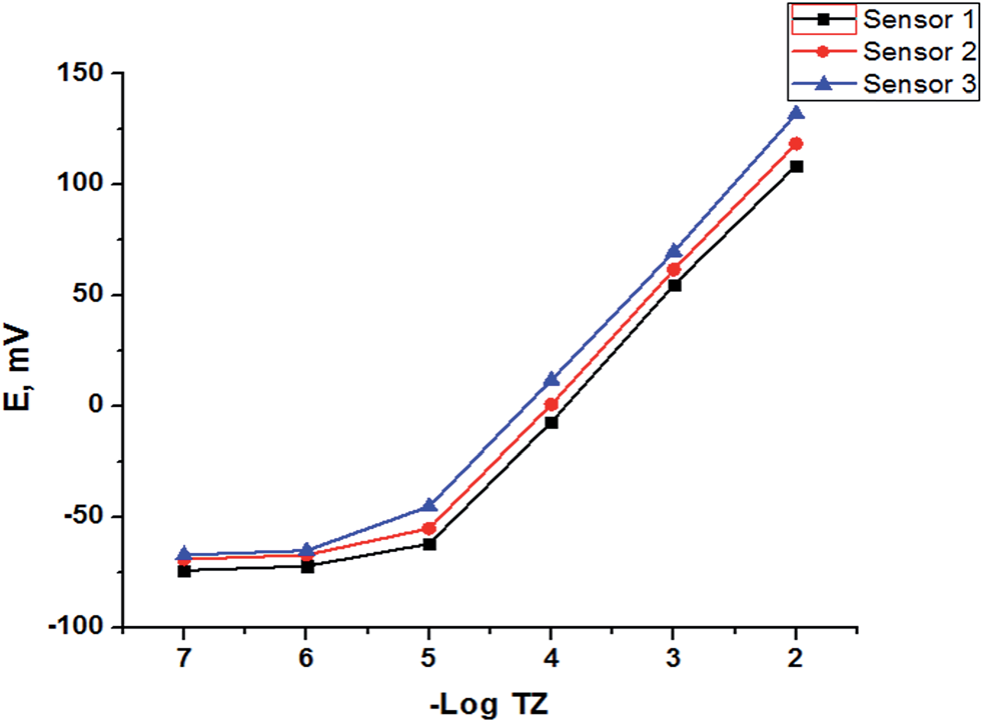
Profile of the potential in mV *versus* −log molar concentration of TZ for different SPEs.

The potentiometric response characteristics of all the SPE sensors were evaluated according to IUPAC recommendations.^37^ Data obtained (Table 2) indicated that the developed sensors can be successfully applied to the potentiometric determination of trazodone hydrochloride in the concentration range of 1 × 10^−5^ to 1 × 10^−2^ mol L^−1^ with Nernstian cationic slopes depending on the method of fabrication. The limit of detection was found to be 7.90 × 10^−6^, 7.60 × 10^−6^ and 6.8 × 10^−6^ mol L^−1^ for sensors 1, 2 and 3, respectively, and although the Au NP SPE (sensor 3) gave the best performance rather than the other two electrodes as shown in Fig. 7 and Table 2, sensors 1 and 2 have the advantage of low cost over sensor 3.

**Table 2:** Electrochemical response characteristics of the proposed SPE sensors

Parameter	Bare SPE	Functionalized SPE	Au NP SPE
Slope (mV per decade)	−57.5	−58.3	−59.05
Intercept (mV)	229.33	235.8	249.05
Correlation coefficient	0.9994	0.9998	0.9996
LOD (M)	7.9 × 10^−6^	7.6 × 10^−6^	6.8 × 10^−6^
Response time (sec)	15	15	10
Isothermal coefficient (V °C^−1^)	0.00189	0.00187	0.00184
Working pH range	4–8	4–8	4–8
Concentration range (mol L^−1^)	10^−5^–10^−2^	10^−5^–10^−2^	10^−5^–10^−2^
Stability (months)	5	6	7
Accuracy (% R)	100.78	101.12	99.62
Precision (% RSD)			
Repeatability^a^	1.345	0.987	0.659
Intermediate precision^b^	1.453	0.876	0.531
Robustness^c^ (mean ± % RSD)	99.02 ± 1.026	98.45 ± 1.106	99.32 ± 0.872

a Average of three different concentrations (1 × 10^−4^, 1 × 10^−3^ and 1 × 10^−2^ mol L^−1^) repeated three times within a day.

b Average of three different concentrations (1 × 10^−4^, 1 × 10^−3^ and 1 × 10^−2^ mol L^−1^) repeated three times in three days.

c Variation in method parameters such as the pH of the sample.

### The effect of soaking

**Fig. 8:**
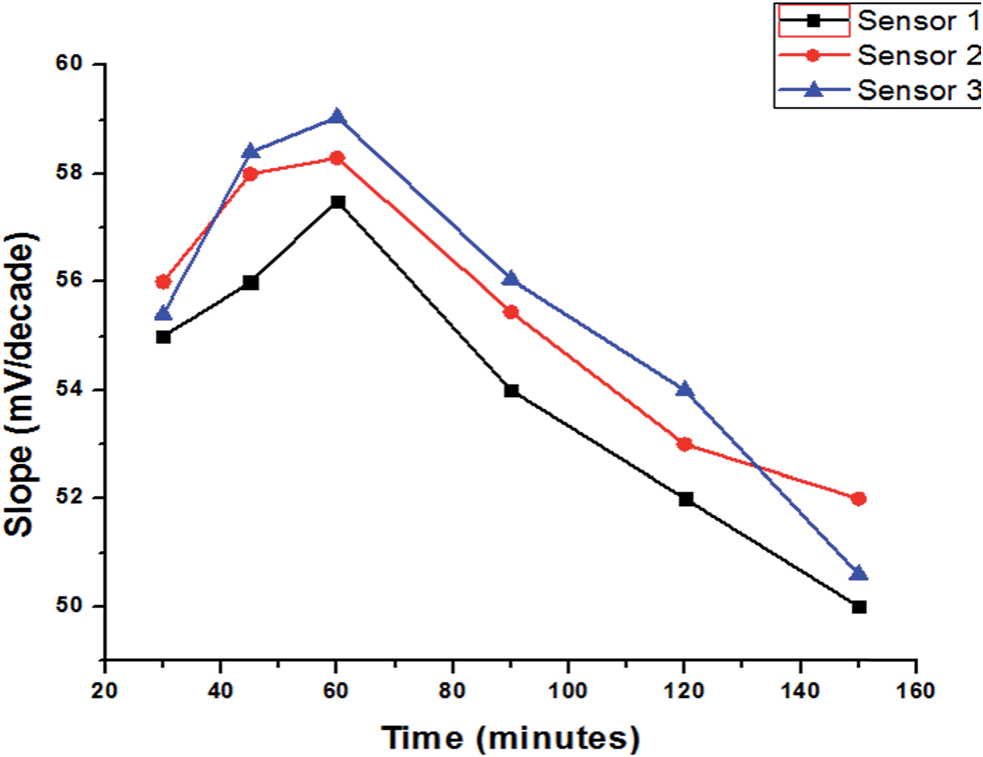
The effect of soaking time on the response of different SPEs.

Activation of the surface membrane of the freshly prepared electrodes is very important to form an infinitesimally thin gel layer at which ion exchange occurs, and that happened by soaking them into stock solution at different times depending on diffusion and equilibration at the interface to obtain the best preconditioning process. The establishment of a fast equilibrium is certainly a sufficient condition to obtain a fast potential response. For the present electrodes, the presoak times were 30, 45, 60, 90, 120 and 150 minutes with optimum slopes 57.5, 58.3 and 59.05 mV per concentration decade for sensor 1, 2 and 3, respectively, and usable concentration ranges of 1 × 10^−5^–1 × 10^−2^, after 60 minutes. Nevertheless, continuous soaking of the electrode in 10^−2^ M trazodone hydrochloride negatively affects its response to the trazodonium cation, which is attributed to leaching of the active ingredients (ion-exchangers, gold nanoparticles and the plasticizer) to the bathing solution. Fig. 8 represents the effect of soaking on the SPEs. However, it was noted that in all cases the electrode which had been kept dry in a closed vessel and stored in a refrigerator showed good preservation of the slope values and response properties extending to several months. Thus, it is recommended that unused electrodes are kept dry in closed vessels in a refrigerator.

### The effect of pH

**Fig. 9:**
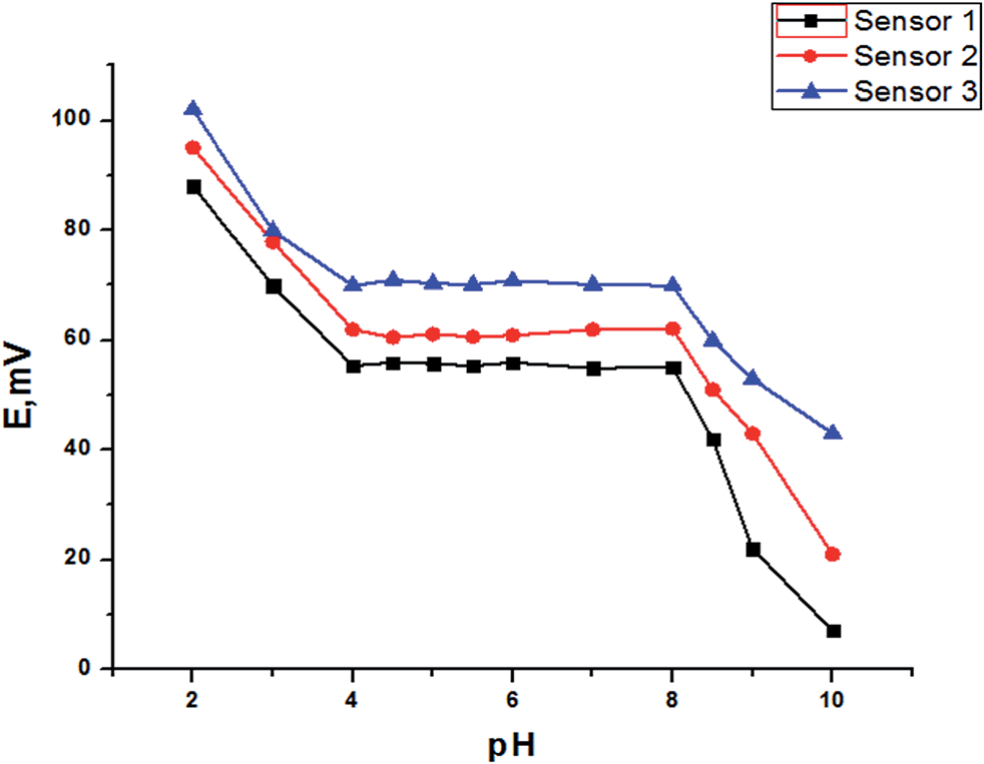
The effect of the pH on the response of different SPEs.

The effect of the pH of the test solution (10^−3^ mol L^−1^ TZ) for SPEs on the electrode potential was investigated. The variation in potential with pH change was followed by addition of small volumes of hydrochloric acid and sodium hydroxide (0.1 mol L^−1^) to the test solutions. Fig. 9 shows the variation in the potential with pH using a test solution (10^−3^ mol L^−1^ TZ) as representative curves. It is evident that the electrode does not respond to pH changes in the range 4.0–8.0. At pH values lower than 4.0 the potential readings increase slightly; the increase in potential is most probably attributed to penetration of the hydronium ions into the membrane surface. At pH values higher than this range, the decrease in the potential readings is attributed either to penetration of hydroxyl ions into the gel layer of the membrane or to deprotonation of TZ in the solution leading to a gradual decrease in its concentration.

### Selectivity

The influence of some inorganic cations, sugars and amino acids on the TZ-electrode was investigated. The selectivity coefficients 
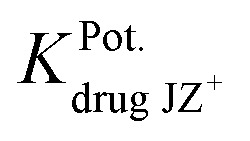
 have been determined by the separate solution method for inorganic cations using the following equation:^45^
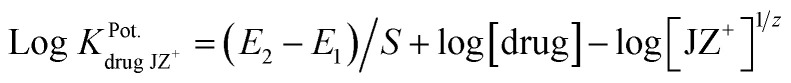
where *E*_1_ and *E*_2_ are the electrode potentials of the 1.0 × 10^−3^ mol L^−1^ solution of each of the investigated drug and interferent cation [JZ^+^], respectively, and *S* is the slope of the calibration graph.

Meanwhile, for sugars and amino acids this is determined using the matched potential method using the following equation:^46^*k*^Pot.^_P,I_ = (aP′ − aP)/aIwhere a known activity (aP′) of the primary ion solution is added into a reference solution that contains a fixed activity (aP) of primary ions and the corresponding potential change (Δ*E*) is recorded, and aI is the activity of the interfering ion that produced the same potential change (Δ*E*). The selectivity coefficients obtained show that the proposed electrode is highly selective towards the TZ ion. There is no interference from the inorganic cations due to the differences in their ionic size, mobility and permeability in comparison to the TZ ion. In the case of sucrose, glucose, urea and glycine the selectivity is most probably attributed to the difference in polarity and to the moderately hydrophobic nature of their molecules relative to the TZ ion (Table 3).

**Table 3:** Selectivity coefficients and tolerance values for different SPEs

Interferent^a^ (10^−3^ M)	log *K*					
	SSM^b^			MPM^c^		
	Sensor 1	Sensor 2	Sensor 3	Sensor 1	Sensor 2	Sensor 3
KCl	−2.62	−2.75	−2.82	—	—	—
CaCl_2_	−2.85	−2.95	−3.05	—	—	—
MgCl_2_	−2.42	−2.55	−2.62	—	—	—
NaCl	−2.62	−2.84	−2.87	—	—	—
NiCl_2_·6H_2_O	−3.25	−3.33	−3.55	—	—	—
Glucose	—	—	—	−2.97	−3.36	−3.47
Urea	—	—	—	−2.40	−2.65	−2.70
Glycine	—	—	—	−3.39	−3.57	−3.72
Sucrose	—	—	—	−3.02	−3.11	−3.21
Citric acid	—	—	—	−2.87	−2.96	−3.04

a All interferents are at a concentration of 1 × 10^−3^ mol L^−1^.

b SSM: separate solution method.

c MPM: matched potential method.

### Response time

**Fig. 10:**
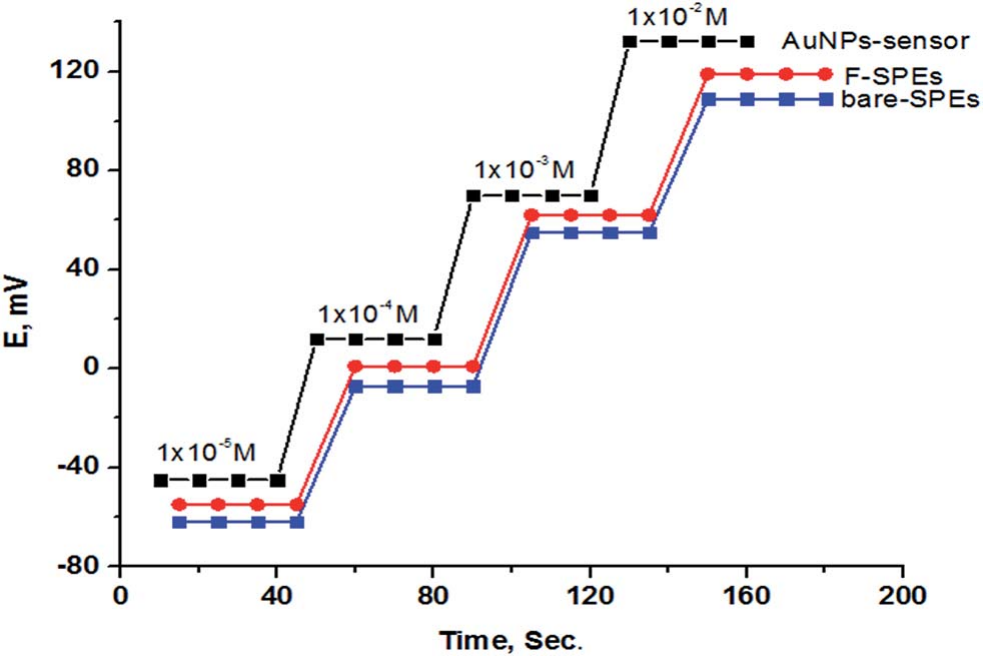
Dynamic response times of the proposed different SPEs.

The dynamic response time of the sensors under study was investigated for the concentration range of 1 × 10^−5^ to 1 × 10^−2^ mol L^−1^. The proposed sensors have short response times of 15 s for sensors 1 and 2 and 10 s for sensor 3 (Fig. 10).

### The effect of temperature

To study the thermal stability of the electrodes, calibration graphs (electrode potential (*E*_elec._) *versus* pTZ) were constructed at the test solution temperatures (25, 30, 35, 40 and 45 °C). The standard electrode potential (*E*^o^) at different temperatures was obtained from the calibration graphs as the intercept at pTZ = 0 and plotted *versus t* − 25, where *t* is the temperature of the test solution in °C. A straight line plot was obtained using the following equation:^47^*E*^o^ = *E*^o^(25) + (d*E*^o^/d*t*)(*t* − 25)

The slope of the straight line obtained represented the isothermal coefficient of the electrode (d*E*^o^/d*t*) and was 0.00189, 0.00187 and 0.00184 V °C^−1^ for sensor 1, 2 and 3, respectively; the small value of d*E*^o^/d*t* for all the sensors indicated a fairly high thermal stability within the temperature ranges investigated (supplied in ESI as Fig. S1 and S2†).

### Storage stability

Although the electrode is a disposable device, a longer stability test was also carried out and the electrodes were successfully used for at least 30 consecutive measurements. The average lifetime for the bare and functionalized SPEs was in the range of 5–6 months while the AuNP-SPEs applied here were tested for a period of 8 months, during which the electrode was used extensively (1 hour per day) and the electrode was stable for about 7 months. After this time the slope of the sensors decreased with a slight gradual decrease in the slope (from 59.05 to 54.88 mV per decade) and the detection limit increased (from 6.8 × 10^−6^ mol L^−1^ to 8.8 × 10^−6^ mol L^−1^). The reason for the limited lifetimes of the SPEs can be attributed to one of the following factors, namely: the loss of plasticizer, carrier, or ionic sites from the polymeric film due to leaching into the sample.

## Method validation

The analytical method was validated according to international conference on harmonization (ICH) guidelines.

### Linearity

Under the described experimental conditions, the calibration graph for the method was constructed by plotting the e.m.f. *versus* drug concentration in moles per litre. The regression plot was found to be linear over the range 10^−5^ –10^−2^ mol L^−1^ and the linear regression equations for the graph are:*E* = −57.50 log[*C*] + 225 (*r* = 0.9994), sensor 1*E* = −58.30 log[*C*] + 235.8 (*r* = 0.9998), sensor 2*E* = −59.05 log[*C*] + 249.05 (*r* = 0.9996), sensor 3where *E* is the potential difference, *C* is the drug concentration in moles per litre and *r* is the correlation coefficient. The linearity ranges, regression equations, intercepts, slopes and correlation coefficients for the calibration data are summarized in Table 2.

### Limit of detection (LOD)

The LOD was calculated according to IUPAC recommendations^48^ from the intersection of the two extrapolated linear portions of the curve in Fig. 7. The values listed previously in Table 2 indicate that the proposed Au NP SPE sensor was more sensitive than sensors 1 and 2 for detection of low concentrations of TZ.

### Accuracy

The accuracy of the proposed SPE sensors for the determination of TZ was investigated using standard addition and direct methods. The results summarized in Tables 2 and 4 show that the proposed method is an accurate one, as indicated by the percentage recovery values, for the determination of TZ in its pharmaceutical preparations without interference from co-formulated additives.

**Table 4:** The determination of TZ in pure solutions using the standard addition technique

Pure added solution (mol L^−1^)	Taken (mol L^−1^)	Sensor 1		Sensor 2		Sensor 3	
		Found (mol L^−1^)	% recovery	Found (mol L^−1^)	% recovery	Found (mol L^−1^)	% recovery
1 × 10^−5^	1 × 10^−2^	1.0098 × 10^−5^	100.98	0.9967 × 10^−5^	99.67	1.0053 × 10^−5^	100.53
5 × 10^−5^		4.934 × 10^−5^	98.68	5.032 × 10^−5^	100.64	4.9825 × 10^−5^	99.65
1 × 10^−4^		1.0008 × 10^−4^	100.08	0.9848 × 10^−4^	98.48	0.9808 × 10^−4^	98.08
5 × 10^−4^		5.0545 × 10^−4^	101.09	4.9335 × 10^−4^	98.67	5.0345 × 10^−4^	100.69
1 × 10^−3^		0.983 × 10^−3^	98.33	0.9965 × 10^−3^	99.65	0.9945 × 10^−3^	99.45
Mean ± S.D.		99.83 ± 1.279		99.42 ± 0.873		99.68 ± 1.043	
% RSD		1.281		0.878		1.046	

### Precision

In order to determine the precision of the proposed method, solutions containing three different concentrations of TZ were prepared and analyzed in 3 replicates and the analytical results are summarized in Table 2. The low values of the relative standard deviation (% RSD) also indicate the high precision of the proposed method. % RSD values were obtained within the same day to evaluate the repeatability (intra-day precision) and over three days to evaluate intermediate precision (inter-day precision).

### Robustness

The robustness of the proposed method was tested by investigating to what extent the capacity of the method remains unaffected by a small deliberate variation in the method parameter conditions such as pH (±0.1) and soaking time (±2 min); in each case only one parameter was changed while all other conditions were kept constant and no marked changes were observed in the results, confirming the robustness of the procedure.^49,50^

### Analytical applications

The optimized sensor under investigation has been successfully used for the potentiometric determination of TZ (pure form and pharmaceutical form) using the standard addition method and the results are summarized in Table 4. In order to estimate the quality of the results, the mean unknown concentration, *C*_*x*_, the mean recovery and the relative standard deviation values were also determined and are represented in the same table. These results showed that the proposed sensor has good efficiency in terms of sensitivity and can be used successfully for quality control of the TZ drug in pure and pharmaceutical preparations. Also, the result obtained from pharmaceutical determination was compared to that from the USP official method^3^ and the data obtained are summarized in Table 5. Statistical evaluation of the results of analysis of TZ in Trittico® tablets by the proposed sensors and the USP official method^3^ showed that there is no significant difference between the proposed and official method in terms of *F*- and *t*-test values (Table 5).

**Table 5:** Statistical comparison of the results obtained by the proposed SPE sensors and the official method^a^

Taken (mol L^−1^)	Sensor 1		Sensor 2		Sensor 3		Official method		
	Found (mol L^−1^)	% recovery	Found (mol L^−1^)	% recovery	Found (mol L^−1^)	% recovery	Taken (μg mL^−1^)	Found (μg L^−1^)	% recovery
1 × 10^−5^	0.9823 × 10^−5^	98.23	0.9913 × 10^−5^	99.13	1.0023 × 10^−5^	100.23	2	1.991	99.55
5 × 10^−5^	5.057 × 10^−5^	101.14	4.942 × 10^−5^	98.84	4.917 × 10^−5^	98.34	4	4.0032	100.08
1 × 10^−4^	0.986 × 10^−4^	98.60	0.9945 × 10^−4^	99.45	0.99 × 10^−4^	99.00	6	5.887	98.12
5 × 10^−4^	4.988 × 10^−4^	99.76	4.982 × 10^−4^	99.64	4.9325 × 10^−4^	98.65	8	7.9086	98.87
1 × 10^−3^	1.0022 × 10^−3^	100.22	1.0087 × 10^−3^	100.87	1.011 × 10^−3^	101.10	10	9.909	99.09
Mean ± S.D.	99.59 ± 1.189		99.58 ± 0.780		99.46 ± 1.162		99.14 ± 0.736		
% RSD	1.194		0.783		1.168		0.742		
Variance	1.414		0.609		1.350		0.541		
Student’s *t*-test	0.716 (2.306)^b^		0.925 (2.306)^b^		0.523 (2.306)^b^				
*F*-value	2.610 (6.338)^b^		1.123 (6.338)^b^		2.493 (6.338)^b^				

a Official method (HPLC method using an octadecylsilane column and a methanol–0.01 mol L^−1^ ammonium phosphate buffer at pH 6.0 (60 : 40) as the mobile phase).

b The values in parentheses are the corresponding tabulated *t* and *F* values at *P* = 0.05.

## Conclusion

The proposed potentiometric sensors using screen printed electrodes (SPEs) provide an accurate, precise and selective method for the direct determination of trazodone hydrochloride in pure and dosage form without prior separation or derivatization steps.

Gold nanoparticle dependent electrodes have excellent biocompatibility and display magnetic, catalytic, and unique structural, optical and electronic properties, which have made them a very attractive material for chemical sensors. Also, the other sensors show good performance and are low in cost in comparison to the gold nanoparticle sensor. The present work led us to conclude this despite the accuracy of the previously reported methods for trazodone hydrochloride determination.

## Conflicts of interest

There are no conflicts to declare.

## Supplementary Material

RA-008-C8RA00745D-s001

## References

[cit1] David B. and Beringer P., RemCington: The Science and Practice of Pharmacy, Lippincott Williams & Wilkins, 21th edn, 2006

[cit2] British Pharmacopoeia, Her Majesty’s Stationery Office, London, 1998, p. 1318

[cit3] The United States Pharmacopeia, United States Pharmacopoeial Convention, Inc., Twinbrook Parkway, Rockville, MD, Asian edn, 24 revision, 2000, vol. 1681–1682, pp. 2149–2152

[cit4] Ayad M. M., Shalaby A., Abdellatef H. E., Hosny M. M. (2003). Anal. Bioanal. Chem..

[cit5] Harikrishna K., Kumar K. S., Seetharamappa J., Manjunatha D. H. (2006). J. Serb. Chem. Soc..

[cit6] Mohamed G. G., Nour El-Dien F. A., Khalil S. M., Mohamed N. A. (2006). Spectrochim. Acta, Part A.

[cit7] Mohamed G. G., Nour El-Dien F. A., Mohamed N. A. (2007). Spectrochim. Acta, Part A.

[cit8] Subbarao J., Rambabu R., Vidyadhara S., Ram D. J., Tejaswini K. (2015). International Journal of Biological & Pharmaceutical Research.

[cit9] Salama F. M., Attia K. A., Said R. A., EL-Olemy A., Abdel-Raoof A. M. (2017). Int. J. Pharm. Sci. Res..

[cit10] Khalil S. M. (1999). Analyst.

[cit11] Ammar R., Khalaf N., Al-Warthan A. (2011). J. Inclusion Phenom. Macrocyclic Chem..

[cit12] Kauffmann J. M., Vire J. C., Patriarche G. J., Nunez-Vergara L. J., Squella J. A. (1987). Electrochim. Acta.

[cit13] Dogrukol-Ak D., Zaimoglu V., Tuncel M. (1999). Eur. J. Pharm. Sci..

[cit14] Hegde R. N., Shetti N. P., Nandibewoor S. T. (2009). Talanta.

[cit15] Azeem I., Mohiuddin S., Fatima A. (2016). J. Basic Appl. Sci..

[cit16] Lovett L. J., Nygard G. A., Khalil S. K. W. (1987). J. Liq. Chromatogr..

[cit17] Mercolini L., Colliva C., Fanali M. S., Raggi M. A. (2008). J. Pharm. Biomed. Anal..

[cit18] Sane R. T., Nerurkar V. R., Tendolkar R. V., Gangal D. P., Mainkar P. S., Dhumal S. N. (1990). Indian Drugs.

[cit19] Ohkubo T., Osanai T., Sugawara K., Ishida M., Otani K., Mihara K., Yasui N. (1995). J. Pharm. Pharmacol..

[cit20] Vatassery G. T., Holden L. A., Hazel D. K., Dysken M. W. (1997). Clin. Biochem..

[cit21] El Gindy A. E., Farouk M., Abd El Aziz O., Abdullah E. A. (2009). J. Appl. Sci. Res..

[cit22] Anderson W. H., Archuleta M. M. (1984). J. Anal. Toxicol..

[cit23] Patel B. N., Sharma N., Sanyal M., Pranav S., Shrivastava J. (2008). J. Chromatogr. B: Anal. Technol. Biomed. Life Sci..

[cit24] Kale P., Agrawal Y. K., Gupta S., Patel C., Patel I. (2014). Int. J. Pharmacol. Pharm. Sci..

[cit25] Mennickent S., González A., Vega M., Ríos G., De diego M. (2014). J. Chil. Chem. Soc..

[cit26] El-Gindy A., El-Zeany B., Awad T., Shabana M. M. (2001). J. Pharm. Biomed. Anal..

[cit27] Yang G. J., Liu P., Qu X. L., Shen M., Wang C. Y., Qu Q. S., Hu X. Y., Leng Z. Z. (2007). Anal. Lett..

[cit28] Kadam P. M., Tarwal N. L., Mali S. S., Deshmukh H. P., Patil P. S. (2011). Enhanced electrochromic performance of f-MWCNT-WO_3_ composite. Electrochim. Acta.

[cit29] Pisal S. H., Harale N. S., Bhat T. S., Deshmukh H. P., Patil P. S. (2014). IOSR J. Appl. Chem..

[cit30] Amjadi M., Samadi A. (2011). J. Food Drug Anal..

[cit31] Park Y. Y., Im A. R., Hong Y. N., Kim C. K., Kim Y. S. (2011). J. Nanosci. Nanotechnol..

[cit32] Apyari V. V., Dmitrienko S. G., Arkhipova V. V., Atnagulov A. G., Zolotov Y. A. (2012). Anal. Methods.

[cit33] Hormozi-Nezhad M. R., Seyedhosseini E., Robatjazi H. (2012). Sci. Iran., Trans. F.

[cit34] Khaled E., Mohamed G. G., Awad T. (2008). Sens. Actuators, B.

[cit35] Khaled E., Hassana H. N. A., Girgis A., Metelka R. (2008). Talanta.

[cit36] Mohamed G. G., Ali T. A., El-Shahat M. F., Migahed M. A., Al-Sabagh A. M. (2012). Drug Test. Anal..

[cit37] Buck R. P., Lindner E. (1994). Pure Appl. Chem..

[cit38] Nour El-Dien F. A., Mohamed G. G., Frag E. Y. Z., Mohamed M. E.-B. (2012). Int. J. Electrochem. Sci..

[cit39] Ali T. A., Mohamed G. G., Omar M. M., Abdrabou V. N. (2015). Int. J. Electrochem. Sci..

[cit40] Ensafi A. A., Doozandeh F., Allafchian A. R. (2010). J. Braz. Chem. Soc..

[cit41] Mohammad-khah A., Ansari R., Delavar A. F., Mosayebzadeh Z. (2012). Bull. Korean Chem. Soc..

[cit42] Arkan E., Saber R., Karimi Z., Mostafaie A., Shamsipu M. (2014). J. Pharm. Biomed. Anal..

[cit43] Guilbault G., Drust R. A., Frant M. S. (1976). Recommendations for nomenclature of ion-selective electrodes. Pure Appl. Chem..

[cit44] Ou Y. Y., Huang M. H. (2006). J. Phys. Chem. B.

[cit45] Frant S. M., Ross J. J. (1968). Anal. Chem..

[cit46] Bassett J. , Denny R. C. and Jeffrey J. M., Text book of Quantitative Inorganic Analysis, Vogel, 4th edn, 1978

[cit47] Antropov L. L. , Theoretical Electrochemistry, Mir, Moscow, 1977

[cit48] Analytical Chemistry Division IUPAC (1994). Recommendation for Nomenclature of Ion Selective Electrode. Pure Appl. Chem..

[cit49] The United States Pharmacopoeia , National Formulary, United States Pharmacopoeia Convention, Rockville, MD, USA, 25th edn, 2007

[cit50] El-Tohamy M., Razeq S., El-Maamly M., Shalaby A. (2010). Cent. Eur. J. Chem..

