# Supplementary material for: Disposable gold nanoparticle functionalized and bare screen-printed electrodes for potentiometric determination of trazodone hydrochloride in pure form and pharmaceutical preparations
Source: RSC Adv. 2018 Mar 23;8(21):11517–27. doi: 10.1039/c8ra00745d (PMC9079154; doi:10.1039/c8ra00745d)
Supplement: RA-008-C8RA00745D-s001 [file RA-008-C8RA00745D-s001.pdf]

Electronic Supplementary Material (ESI) for RSC Advances.
This journal is © The Royal Society of Chemistry 2018

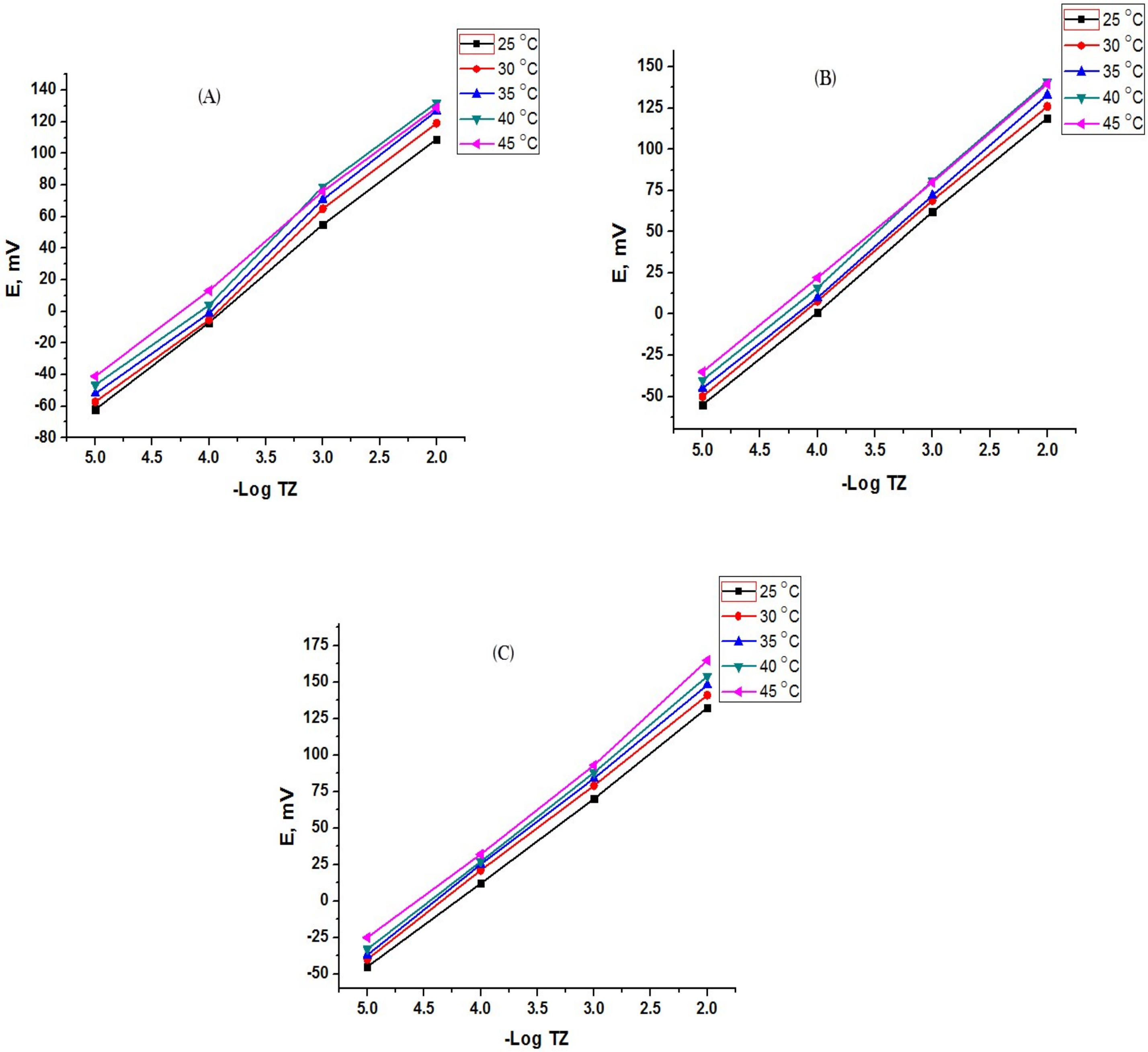

Figure (S1) calibration curves of A) sensor 1 B) sensor 2 and C) sensor 3 at different temperatures.

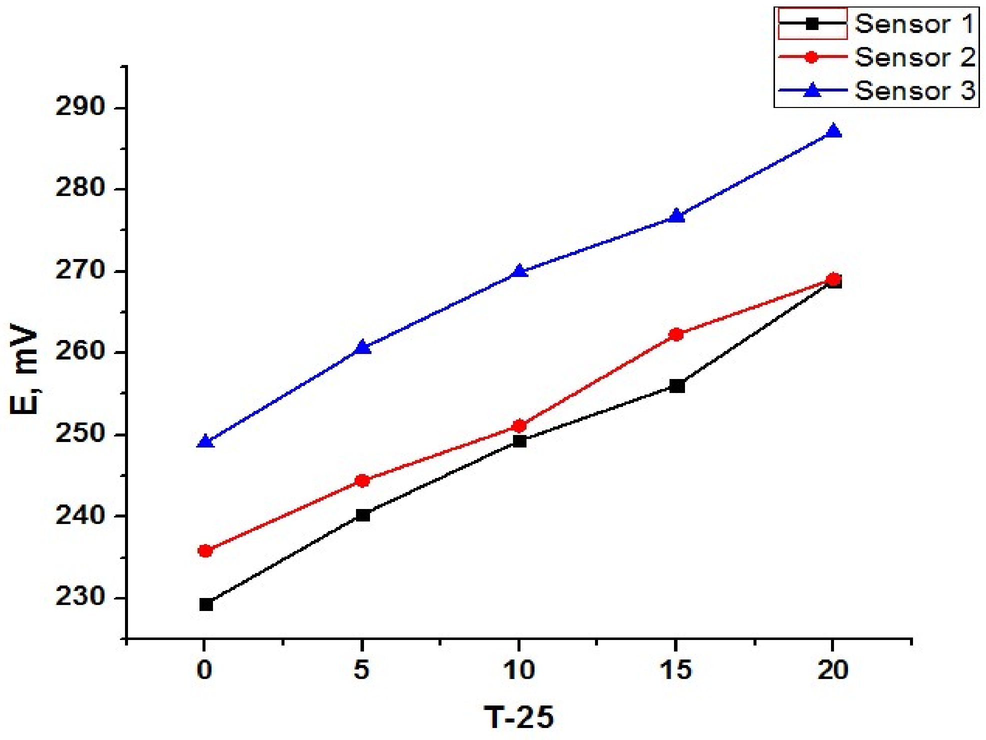

Figure (S2): Variation of standard electrode potential with changes of test solution temperatures for different SPEs.
